# Co-Delivery of Nano-Silver and Vancomycin via Silica Nanopollens for Enhanced Antibacterial Functions

**DOI:** 10.3390/antibiotics11050685

**Published:** 2022-05-18

**Authors:** Chengang Ni, Yuening Zhong, Weixi Wu, Yaping Song, Pooyan Makvandi, Chengzhong Yu, Hao Song

**Affiliations:** 1 Australian Institute for Bioengineering and Nanotechnology, The University of Queensland, Brisbane 4072, Australia; chengang.ni@uq.net.au (C.N.); yuening.zhong@uq.net.au (Y.Z.); weixi.wu@uq.net.au (W.W.); yaping.song@uq.net.au (Y.S.); c.yu@uq.edu.au (C.Y.); 2 Center for Materials Interfaces, Istituto Italiano di Tecnologia, Pontedera, 56025 Pisa, Italy; pooyan.makvand@iit.it

**Keywords:** nanomaterials, antibacterial, silica nanoparticles, silver nanoparticles, vancomycin, drug delivery

## Abstract

Infectious diseases caused by bacteria have led to a great threat to public health. With the significant advances in nanotechnology in recent decades, nanomaterials have emerged as a powerful tool to boost antibacterial performance due to either intrinsic bactericidal properties or by enhancing the delivery efficiency of antibiotics for effective pathogen killing. Vancomycin, as one of the most widely employed antimicrobial peptides, has a potent bactericidal activity, but at the same time shows a limited bioavailability. Silver nanoparticles have also been extensively explored and were found to have a well-recognized antibacterial activity and limited resistance potential; however, how to prevent nanosized Ag particles from aggregation in biological conditions is challenging. In this study, we aimed to combine the advantages of both vancomycin and nano-Ag for enhanced bacterial killing, where both antibacterial agents were successfully loaded onto a silica nanoparticle with a pollen-like morphology. The morphology of nano-Ag-decorated silica nanopollens was characterized using transmission electron microscopy and elemental mapping through energy dispersive spectroscopy. Silver nanoparticles with a size of 10–25 nm were observed as well-distributed on the surface of silica nanoparticles of around 200 nm. The unique design of a spiky morphology of silica nano-carriers promoted the adhesion of nanoparticles towards bacterial surfaces to promote localized drug release for bacterial killing, where the bacterial damage was visualized through scanning electron microscopy. Enhanced bactericidal activity was demonstrated through this co-delivery of vancomycin and nano-Ag, decreasing the minimum inhibition concentration (MIC) towards *E. coli* and *S. epidermidis* down to 15 and 10 µg/mL. This study provides an efficient antimicrobial nano-strategy to address potential bacterial infections.

## 1. Introduction

Bacterial infections pose severe threats to the public health, especially given the ever-increasing level of drug resistance due to the overuse of antibiotics. Nowadays, infectious diseases are even more difficult to treat through conventional medications, which may cause 10 million deaths per year by 2050, overtaking cancer and heart diseases as the leading cause of death in humans [1,2]. To address this challenge, various strategies have been developed to combat bacterial infection with an efficient pathogen-killing effect and reduced resistance [3,4]. For instance, as safe alternatives to conventional synthetic antibiotics, natural antimicrobial compounds, such as essential oils extracted from plants [5], enzymes derived from animal sources [6], bacteriocins obtained from pathogens [7], and naturally occurring peptides and polymers [8,9] were favorably chosen for infection treatment. In addition, remarkable advancements in nanotechnology enabled the development of antibacterial nano-agents, which could either show an intrinsic bactericidal activity or enable the efficient delivery of antimicrobial compounds to kill bacteria [10,11,12].

As a natural metabolite, glycopeptide of vancomycin has been widely explored, showing potent antimicrobial activity, especially for Gram-positive strains [13]. Vancomycin can strongly bind with the D-Ala-D-Ala terminal at the end of the peptide chain during bacterial cell-wall synthesis, thus inhibiting the crosslinks that form and prevent the bacteria from building an intact peptidoglycan layer [14]. To this end, vancomycin has been used for bacterial inhibition and, at the same time, served as a targeting ligand to bind with the bacterial surface for efficient antibiotics delivery [15,16]. It is noted that >90% of vancomycin can be renally cleared within 24 h of dosing [17]. Therefore, to prolong the circulation half-life and enable a sustained release of vancomycin for long-term bacterial inhibition, various types of nanoparticles were employed to load or encapsulate this hydrophilic glycopeptide. For example, mesoporous carbon nanoparticles were used to enhance the hydrophobic interactions between nanoparticles and vancomycin, where the sustained release was demonstrated by tuning the carbon shell thickness and pore size [18]. Porous silica has also been widely used for antimicrobial compound delivery, but its hydrophilic nature typically renders the quick release of water-soluble drugs. It has been reported that engineering rough textures on silica nanoparticles showed hydrophobic surface properties, which enabled the sustained release of vancomycin [19]. Moreover, the rough surface enhanced bacterial adhesion, which promoted the localized release of vancomycin to achieve efficient antibacterial performance. However, the repeated use of single antibacterial agents such as vancomycin may cause drug resistance to develop [20,21]. It is expected that novel bactericidal nanomaterials with multiple antibacterial functions could allow for efficient bacterial killing and prevent the potential induction of resistance.

As one of the most widely used antibacterial agents that can intrinsically kill bacteria, Ag nanoparticles (nano-Ag) have attracted a significant research interest in the past decades [22,23]. Regarding the antibacterial mechanism of nano-Ag, the leaching of Ag^+^ ions that strongly bind to proteins and nucleic acids, causing cell death, has been widely reported [22,23,24,25]. Moreover, the generation of reactive oxygen species (ROS) has been recognized as another antibacterial path of nano-Ag [24,25]. Recently, intracellular dysfunctions induced by the direct contact or internalization of nano-Ag with bacteria were revealed to be responsible for bacterial killing [25]. This multi-approach bacterial killing reduced the chance of resistance development; thus, many types of nano-Ag were synthesized for antibacterial applications [26,27]. However, when pushing Ag nanoparticles into the dimension of nanometer scale, nano-Ag ensures a high antibacterial property but easily aggregates together, hindering its practical use [28]. Anchoring nanoparticles to a porous support can prevent severe aggregation and allow the efficient delivery of these nano-silver particles to bacteria for the sustained release of Ag^+^ for prolonged antibacterial performance [29,30,31]. Notably, the physicochemical features of support particles such as surface topography were found to play an important role in regulating Ag loading and antibacterial performance. In particular, dendritic and spiky silica nanoparticles facilitate efficient nano-Ag loading and subsequent antibacterial activity [32].

**Scheme 1:**
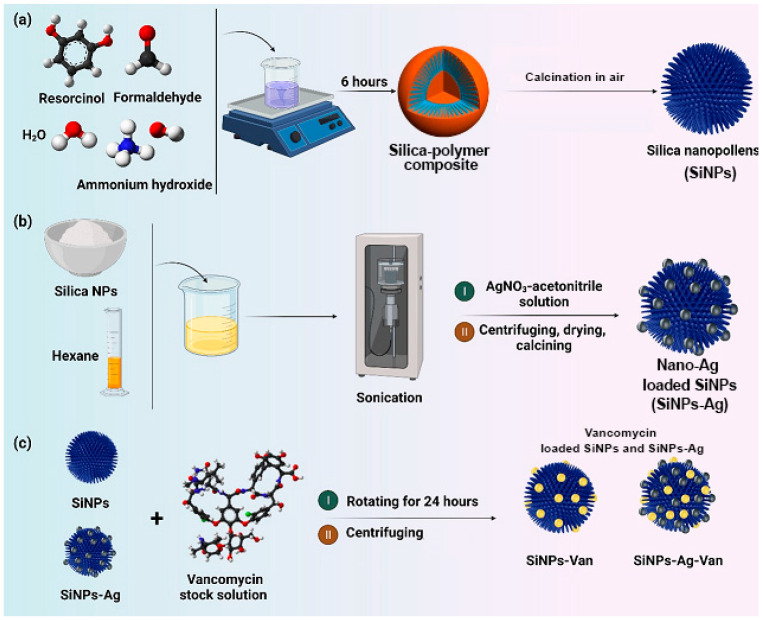
Schematic presentation of the fabrication of silica nanopollens (**a**), decoration of nano-Ag (**b**), and loading of vancomycin in the nanopollen particles (**c**).

In this study, we report the fabrication of pollen-like spiky silica nanoparticles loaded with both antibacterial drugs of vancomycin and nano-silver, denoted as SiNPs-Ag-Van, for enhanced antibacterial applications (Scheme 1). The porous nature of these nanoparticles allowed for a high loading of both cargos. The spiky nanotopography promoted interactions with pathogens for surface adhesion to boost the localized delivery of antibacterial agents. The developed nano-formulation showed a strong bacterial inhibition capability towards both the Gram-positive strain of *S. epidermidis* and Gram-negative bacteria of *E. coli*. The spiky silica nanoparticle-enabled co-delivery platform provides a promising approach in combatting bacterial infection with a high efficiency and potential low resistance.

## 2. Results

### 2.1. Synthesis and Characterizations of SiNPs-Ag

Silica nanoparticles with a pollen-like spiky nanotopography were fabricated according to our previous report through a polymer-silica co-assembly sol–gel approach [33,34]. In brief, a polymer core was first synthesized, followed by the in-situ growth of a silica-polymer interpenetrating a composite layer by controlling the polymer and silica condensation kinetics. Upon high-temperature calcination in air to remove the organic content from the composite, silica nanoparticles with a hollow interior and numerous surface spikes were obtained. 

To decorate nano-Ag nanoparticles on silica surfaces, a dual-solvent strategy was used to allow Ag precursors to reside within the porous space on the silica nanoparticle surface [35]. Typically, hydrophilic silica powder was dispersed into a nonpolar solvent of hexane to wet the particles through sonication. At the same time, the silver precursor, AgNO_3_, was then dissolved into a polar solvent of acetonitrile that was immiscible with hexane. A small volume of the AgNO_3_–acetonitrile solution was then added into the silica–hexane suspension, where by controlling the volume of acetonitrile solution equal to the pore volume of silica nanoparticles (pre-measured through nitrogen sorption analysis, 0.33 cm^3^/g), most of the polar solvent could be infiltrated into the pores of these nanoparticles due to capillary effect. To this end, Ag precursor could be managed so that it resided on the silica surface and was converted to Ag nanoparticles through drying and calcination processes, rather than remaining in the solution and yielding with silver particle aggregates outside of the silica particles [36].

**Figure 1:**
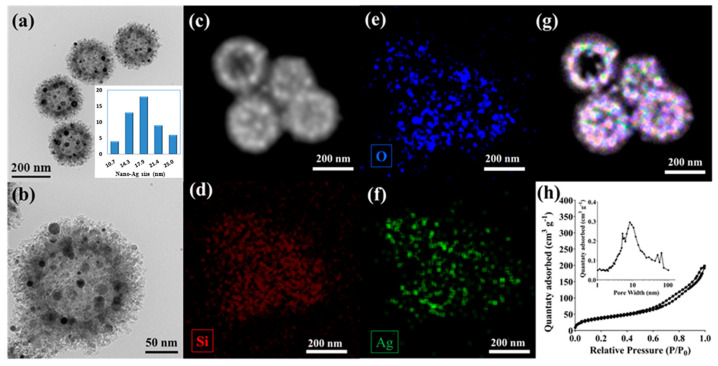
TEM images (**a**,**b**), with nano-Ag particle size distribution as inset of (**a**), dark field image (**c**), corresponding elemental mapping on silicon (**d**), oxygen (**e**), silver (**f**), Si-Ag-O merged image (**g**), and nitrogen sorption isotherm and (**h**), inset of pore size distribution for SiNPs-Ag.

As observed in Figure 1a,b, the transmission electron microscopy (TEM) images demonstrated a successful loading of Ag nanoparticles on spiky silica nanoparticles, denoted as SiNPs-Ag, through the above-mentioned dual-solvent approach. Silica nanoparticles showed a pollen-like spiky surface nanotopography, with a particle size of around 200 nm and a hollow core of ~110 nm. Ag nanoparticles of sizes ranging from 10 to 25 nm were clearly identified as uniformly distributed on the silica particle surface without obvious aggregation. The nanoparticles characterized in a dark field under STEM (Figure 1c) and analyzed through EDS mapping (Figure 1d–g) further confirmed the efficient loading of nano-Ag on silica particles, where the element of Ag overlapped well with the elements of Si and O, indicating a well-dispersed Ag nanoparticle distribution. 

As observed from the microscopy images where a large number of nano-Ag appeared on the porous silica surface, it is critical to understand whether there remains a porous structure with a high surface area to accommodate other antimicrobial agents such as, in this case, vancomycin. Therefore, a nitrogen sorption analysis of the sample was carried out with the N_2_ adsorption–desorption isotherm plotted as Figure 1h, and the pore size distribution is shown in the inset. Clearly, the SiNPs-Ag sample maintained a type-IV isotherm pattern with a mesopore size of around 10 nm. Compared to bare SiNPs without nano-Ag loading, the surface area of SiNPs-Ag decreased from 176 m^2^/g to 132 m^2^/g, while the pore volume remained around 0.3 cm^3^/g.

### 2.2. Vancomycin Loading and Release on SiNPs-Ag

**Figure 2:**
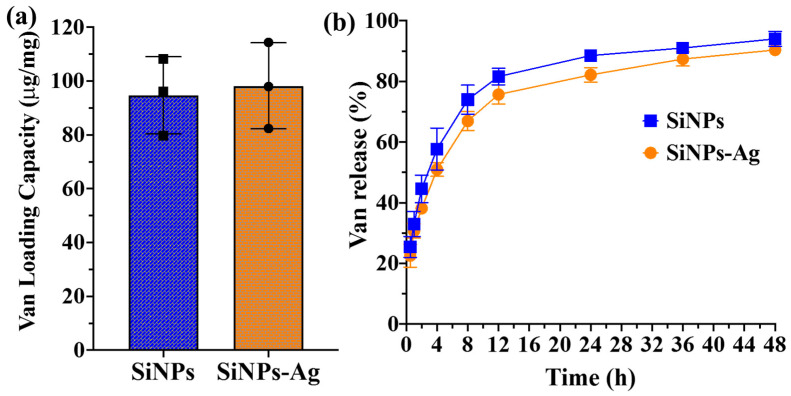
Vancomycin loading capacity (**a**) and release profile in PBS (**b**) for SiNPs and SiNPs-Ag. Error bars represent standard derivation (*n* = 3).

The porous nature of SiNPs-Ag allowed antibacterial molecules of vancomycin to be transported towards the porous silica surface and loaded on the particles. We then mixed vancomycin with both SiNPs and SiNPs-Ag samples in a PBS solution and measured the vancomycin loading capacity and release profile from these nanoparticles. According to Figure 2a, spiky silica nanoparticles with and without nano-Ag showed a similar vancomycin loading capacity of approximately 100 µg/mg. The vancomycin release profiles of these two particles were shown in Figure 2b, where both nanoparticles allowed a sustained release behavior of antibacterial biomolecules, releasing around 50% of vancomycin in the first 4 h and another 40% of the drug in the next 44 h. Notably, no significant difference in both vancomycin loading and release was identified between SiNPs and SiNPs-Ag samples, suggesting that the loading of Ag nanoparticles did not negatively impact the second antibacterial agent loading for pathogen killing.

### 2.3. Antibacterial Performance of SiNPs-Ag-Van Nanoformulation

**Figure 3:**
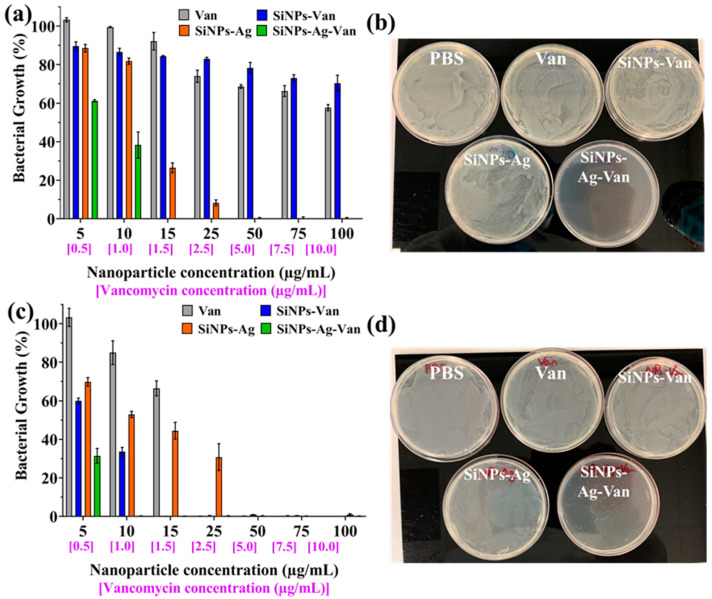
Antibacterial performance against *E. coli* (**a**,**b**) and *S. epidermidis* (**c**,**d**). Error bars represent standard derivations (*n* = 3).

After the successful loading of both nano-Ag and vancomycin on silica nanoparticles to obtain our antibacterial formulation of SiNPs-Ag-Van, its antibacterial performance was then evaluated with both Gram-negative strains of *E. coli* and a Gram-positive strain of *S. epidermidis*. Figure 3a shows the bacterial inhibition profiles of our antibacterial formulations for *E. coli*, with nanoparticles dosed at 5–100 μg/mL and corresponding vancomycin concentrations of 0.5–10 μg/mL. All groups showed a dose-dependent bacterial inhibition performance, while both vancomycin itself and silica nanoparticles loaded with vancomycin (SiNPs-Van) presented a limited antibacterial activity across the dose range. This is due to the fact that vancomycin is not very effective against Gram-negative bacteria. In contrast, the silver-coated nanoparticles (SiNPs-Ag) showed a high bacterial killing rate with a minimum inhibition concentration (MIC) of about 50 μg/mL. Notably, the co-delivery of nano-Ag and vancomycin exhibited a superior bacterial inhibition efficiency, bringing the MIC down to 15 μg/mL. Figure 3b further demonstrates the excellent bactericidal activity of SiNPs-Ag-Van as compared to other formulations when dosed at a nanoparticle concentration of 15 μg/mL. The strong antimicrobial effect by simultaneously delivering silver and vancomycin to Gram-negative bacteria is evident.

Similarly, Figure 3c shows the inhibition profile of *S. epidermidis* in the presence of four types of antibacterial formulations. Notably, there is a clear difference from the results observed in *E. coli*, where vancomycin and vancomycin-containing nanoparticles both showed high bacterial inhibition rates. Van and SiNPs-Van presented an MIC of 25 and 15 µg/mL, respectively, which was much less than the MIC from SiNPs-Ag (~50 µg/mL), while the co-delivery of vancomycin and silver continued to show the best antibacterial performance, with an MIC of 10 µg/mL. Figure 3d provides further evidence regarding the superior bactericidal property of SiNPs-Ag-Van dosed at a nanoparticle concentration of only 10 µg/mL.

### 2.4. Bacterial Morphology Change after Nanoformulation Treatment

**Figure 4:**
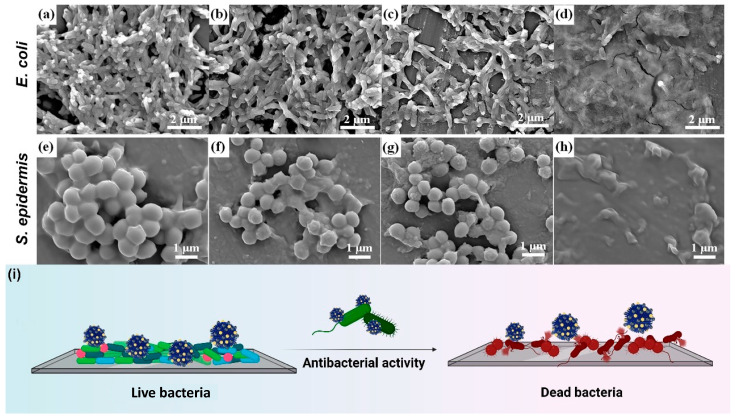
SEM images of *E. coli* (**a**–**d**) and *S. epidermidis* (**e**–**h**) after treatment of vancomycin (**a**,**e**), SiNPs-Van (**b**,**f**), SiNPs-Ag (**c**,**g**) and SiNPs-Ag-Van (**d**,**h**), and schematical illustration of the antibacterial activity (**i**).

By understanding the enhanced antibacterial capability achieved by the co-delivery of nano-Ag and vancomycin using a spiky silica nanoparticle, we further explored how these nanoformulations interacted with bacterial membranes to realize a bacterial killing effect. The morphology of *E. coli* and *S. epidermidis* treated with different antibacterial formulations was characterized by scanning electron microscopy (SEM). As shown in Figure 4a,e, vancomycin itself can hardly induce obvious bacterial damage when dosed lower than its MIC when, as in this case, *E. coli* was treated at 15 µg/mL and *S. epidermidis* was treated at 10 µg/mL. By loading vancomycin into silica nanoparticles, this nano-formulation remained inactive towards the Gram-negative strain of *E. coli* (Figure 4b), which led to some bacterial cell shrinkage/damage for *S. epidermidis*, as observed in Figure 4f. This indicates that the use of silica nanoparticles improves the delivery efficiency of vancomycin towards the bacterial surface, promoting its antibacterial effect, especially against Gram-positive strains. Similarly, nano-Ag-decorated silica nanoparticles caused obvious damage to *E. coli*, rendering cell shrinkage and collapse, as observed in Figure 4c. Evidently, most of the *S. epidermidis* cells maintained their round shapes and remained intact. As shown in Figure 4d,h, it was clear that the co-delivery of nano-Ag and vancomycin can effectively disrupt the bacterial membrane when dosed at their MICs, where no intact bacteria could be identified from images. The bacterial morphology observations under SEM further supported the superior antibacterial property achieved by SiNPs-Ag-Van, as schematically illustrated in Figure 3i.

## 3. Discussion

Developing novel strategies to combat bacterial infections and antibiotic resistance is one of the key research topics that has attracted extensive attention and research efforts. To this end, a wide range of nanomaterials were developed that could directly kill bacterial or enhance the delivery efficiency of existing antibacterial agents for improved performance [12]. This study aimed to combine the use of two widely used antibacterial agents of silver nanoparticles and the antibacterial peptide, vancomycin. To overcome their specific limitations in severe aggregation and short half-life in the body, a silica nano-carrier was used to anchor the nano-sized Ag particles, preventing them from further aggregation. At the same time, the porous nature of the nano-carrier allowed for the efficient loading of vancomycin, enabling the efficient co-delivery of two antibacterial cargos, which has rarely been reported.

Silver nanoparticles have been widely used for antibacterial applications, and various substrate/carriers have been developed to incorporate small-sized Ag particles either inside or on the surface to allow efficient bacterial killing [26,29]. To allow a high loading of Ag and stable immobilization, the silica nanoparticle surface was typically further modified with aminopropyl groups that could form Ag-amine coordination bonds to anchor the nano-Ag [32,37]. In this study, we report a facile Ag loading approach without changing the silica nanoparticle surface chemistry, a dual-solvent method. This method was previously used in catalysts by impregnating the active metal species into the pores of the substrate [35]. In this case, porous silica was first immersed in a unipolar solvent of hexane, followed by the addition of an immiscible polar solvent containing the Ag precursor. The polar solvent preferred adsorption into the nano-sized pores due to capillary effect, which allowed for the precise loading of Ag species on the silica surface. The Ag species were then converted into Ag nanoparticles under high temperature. This simple synthesis strategy enabled high Ag loading on the particle surface, as demonstrated from the TEM images in Figure 1a,b, and more importantly, prevented nano-Ag precipitation outside of the silica nanoparticles.

The loading of nano-Ag did not significantly alter the physicochemical features of silica nanoparticles, providing an abundant surface area and large pore size to accommodate another antimicrobial agent. It was reported that the rough-structured hydrophilic silica nanoparticles showed a relatively hydrophobic surface property, which favored the loading and slow release of vancomycin [19]. The silica nanopollens used in this study shared similar structure and hydrophobic surface properties, as reported in the literature [34], which explains the efficient loading and sustained release profile as shown in Figure 2. The efficient delivery of two antibacterial agents was achieved using pollen-like silica nanoparticles. It was reported that such a rough-structured nanoparticle surface enhanced its adhesion towards bacteria, boosting the local release of cargos. Meanwhile, the surface charge of nanoparticles was also important for bacterial interactions. The surface charge of SiNPs was measured as negative (−16.3 ± 1.7 mV), but was positive (4.9 ± 1.1 mV) after the positively charged nano-Ag loading. By the loading of a cationic antibacterial drug, vancomycin, SiNPs-Ag-Van showed a surface charge of 8.9 ± 0.9 mV, which could also promote their attachment to the negatively charged bacterial surface. Typically, vancomycin is more active against Gram-positive strains when compared to Gram-negative strains, where the outermost cell wall of the peptidoglycan is the major target of vancomycin [16]. In contrast, nano-Ag showed a broader spectrum of bacterial killing, possibly due to its many bacteria-killing mechanisms. This is reflected in the bacterial inhibition results, as shown in Figure 3a,c.

Synergistically, the co-delivery of vancomycin and nano-sized silver particles outperformed each individual antibacterial agent, resulting in a low MIC that required a lower dose of antibacterial formulations to be administrated for efficient bacterial killing, regardless of Gram-positive/negative strains. In general, this co-delivery system has the potential to serve as a promising bactericidal toolkit with a high performance against broad spectrum of strains. 

## 4. Materials and Methods

### 4.1. Synthesis of Silica Nanopollens

Silica nanopollens were synthesized according to our previous reports [33,34]. Typically, 0.15 g of resorcinol and 0.21 mL of formaldehyde (37%) were first dissolved in ethanol (70 mL), water (10 mL), and ammonium hydroxide (28%, 3 mL). Polymer cores were then formed after reacting for 6 h, where silica precursor of tetraethyl orthosilicate (TEOS, 0.6 mL), resorcinol (0.4 g) and formaldehyde (0.56 mL) were sequentially added into the reaction solution. Upon another reaction of 2 h, the precipitates were collected under centrifugation, dried overnight at 50 °C in the oven, and calcined in air at 550 °C for 5 h.

### 4.2. Nano-Ag Loading on SiNPs via Dual-Solvent Method

Silica powder obtained from the above method was then used for Ag nanoparticle loading. Typically, 30 mg of silica nanoparticles was mixed with 2 mL of hexane under sonication, followed by stirring for 4 h under room temperature to allow for the efficient wetting of the silica surface by the solvent. Meanwhile, 2M AgNO_3_–acetonitrile solution was prepared, with only 10 µL of the solution added into the silica-hexane suspension for another stirring of 6 h. The Ag-loaded nanoparticles were then centrifuged, dried, and calcined at 500 °C, leading to the grey nanoparticle powder of SiNPs-Ag.

### 4.3. Characterizations

The nanostructure and composition of SiNPs-Ag were characterized by TEM using a JEOL HT-7700, with a setting voltage of 120 kV. The energy-dispersive X-ray spectroscopy (EDS) integrated in the HT-7700 microscope was used for elemental mapping of the particles under scanning transmission electron microscopy (STEM) mode. Nitrogen adsorption–desorption analysis was carried out by using Micrometrics Tristar 3020II. Before analysis, samples were first degassed overnight under vacuum at 150 °C. The zeta potential of nanoparticles in phosphate-buffered saline (1× PBS, pH = 7.4) was measured by a Zetasizer Nano (Malvern, Worcestershire, UK).

### 4.4. Vancomycin Loading and Release

Vancomycin stock solution was prepared by dissolving 10 mg of vancomycin into 10 mL of PBS (pH = 7.4), achieving a concentration of 1 mg/mL. Silica nanopollens as well as SiNPs-Ag were also suspended in PBS at the same concentration, followed by the mixing of 1 mL of silica suspension with 1 mL of vancomycin stock solution by rotating for 24 h. SiNPs-Ag-Van was then centrifuged down, with the free vancomycin concentration in the supernatant measured using a UV–vis spectrophotometer to calculate the vancomycin loading capacity. To obtain the vancomycin release profile, SiNPs-Ag-Van and SiNPs-Van were both re-suspended into PBS by shaking under 37 °C over two days. A small volume of samples was then collected and centrifuged with the vancomycin content in the supernatant quantified using a UV–vis spectrophotometer (UV-2450, Shimadzu, Kyoto, Japan).

### 4.5. Bacterial Inhibition Assay

Gram-negative bacteria, *E. coli*, and Gram-positive strain, *S. epidermidis*, were first cultured overnight in a LB broth at 37 °C prior to antibacterial assays. Cultures were then diluted with LB to reach a final concentration of 5 × 10^7^ CFU/mL. Samples of Van, SiNPs-Van, SiNPs-Ag, and SiNPs-Ag-Van were prepared at a set of concentrations ranging from 50 to 1000 μg/mL (nanoparticle) and corresponding vancomycin dosages of 5 to 100 μg/mL. Then, 100 μL of antibacterial formulation was mixed with 100 μL of the bacterial solution, followed by topping up the volume to 1 mL with LB medium. The treatment was carried out at 37 °C in a shaker with a constant agitation at 200 rpm for 24 h. The bacterial inhibition capability was then assessed by measuring the absorbance at 600 nm (OD600), where the background absorbance from liquid and formulations without bacterial was also recorded and extracted for a turbidity analysis to assess bacterial inhibition. The bacterial viability was further examined by LB-agar plate assay, where 50 μL of bacteria samples was spread on the LB-agar plate for overnight culture. For *E. coli*, the bacteria were treated with formulations at a nanoparticle concentration of 15 μg/mL (vancomycin at 1.5 μg/mL). For *S. epidermidis*, the bacteria were treated with formulations at a nanoparticle concentration of 10 μg/mL (vancomycin at 1.0 μg/mL). Photographs were taken to identify bacterial colony growth after antibacterial treatments.

### 4.6. SEM Characterizations of Treated Bacteria

To further investigate the bacterial killing effect by using individual antibacterial agents and co-delivery of nano-Ag and vancomycin, a JEOL JSM 7800 was used to characterize the bacterial morphology differences/change after treatment. *E. coli* were treated with formulations at a nanoparticle concentration of 15 μg/mL (vancomycin at 1.5 μg/mL), and *S. epidermidis* were treated with formulations at a nanoparticle concentration of 10 μg/mL (vancomycin at 1.0 μg/mL). After 24 h of treatment, bacteria were fixed by glutaraldehyde and stained by osmium tetroxide (OsO_4_) according to protocol reported in the literature for following SEM characterization [34].

## 5. Conclusions

In summary, a co-delivery system for vancomycin and nano-sized silver was developed by using silica nanopollens as carriers. Firstly, a dual-solvent method was developed to allow the synthesis of nano-Ag decorated silica nanopollen particles, followed by the loading of vancomycin to these functional particles. The vancomycin release profile from the silica nanoparticles showed a sustained release over 2 days, which promoted the bactericidal activity of developed formulations via the localized release of both vancomycin and Ag. The co-delivery of vancomycin and nano-sized silver particles enhanced their bacterial killing performance, compared to the single component of antibacterial agents. Potent antibacterial activity was observed for both Gram-positive and Gram-negative pathogens. An SEM analysis of the bacterial morphology after antibacterial treatment further validated the enhanced nanoparticle–cell interactions that promoted the co-delivery of antibacterial agents efficiently to the bacteria. In general, a new antibacterial co-delivery system has been established with promising antibacterial applications. The new findings and knowledge derived from this work will pave the way for the rational design of antibacterial nanomaterials.

## Data Availability

The data presented in this study are available on request from the corresponding author.
